# Supplementary material for: DropBlot: single-cell western blotting of chemically fixed cancer cells
Source: Nat Commun. 2024 Jul 13;15:5888. doi: 10.1038/s41467-024-50046-0 (PMC11246512; doi:10.1038/s41467-024-50046-0)
Supplement: Supplementary file 1 — Supplementary Information [file 41467_2024_50046_MOESM1_ESM.pdf]

Supplementary Information

DropBlot: single-cell western blotting of chemically fixed cancer cells

Yang Liu[1, 3, *], Amy E. Herr[1, 2, *]

[1]Department of Bioengineering, University of California, Berkeley, California 94720, USA

[2]Chan Zuckerberg Biohub, San Francisco, California 94158

[3]School of Chemical, Materials and Biomedical Engineering, University of Georgia, Athens, Georgia 30602, USA

* Email: liuy@uga.edu, aeh@berkeley.edu

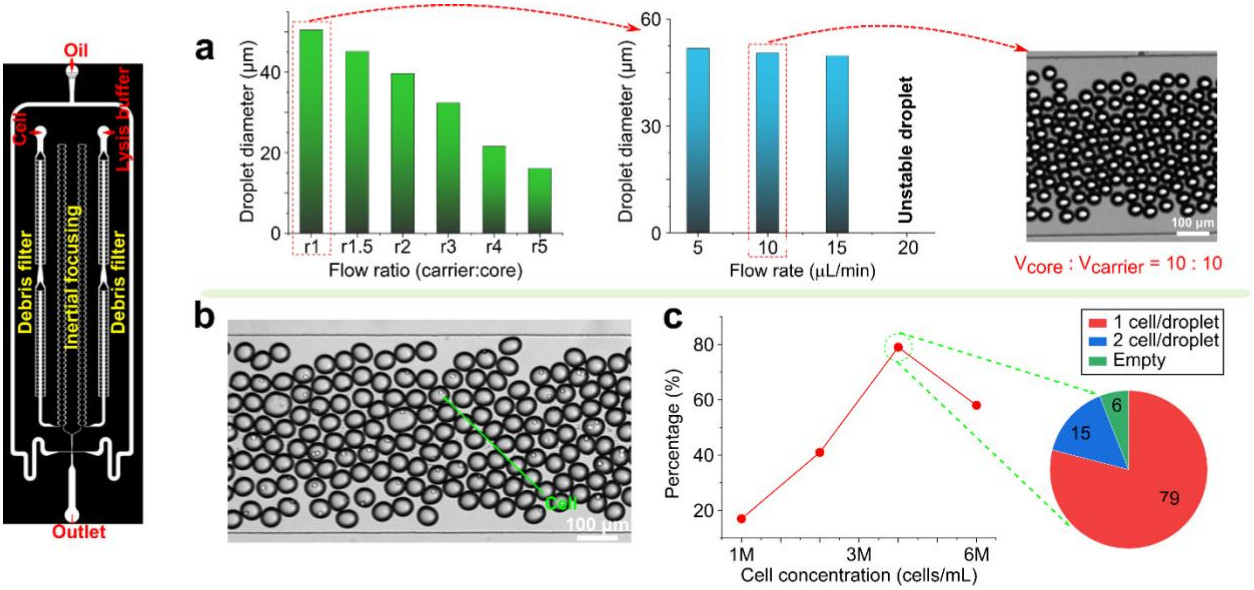

Supplementary Figure 1. Optimization of droplet generation for cell encapsulation. (a) Study of droplet size with different parameters: flow ratio between carrier and core medium (left); flow rate of core medium when the flow ratio was 1 (middle). Stable droplets of 50 μm in diameter were generated (right). n = 300 droplets. (b) Bright-field images of cell encapsulation when the initial concentration was 3 M (3.0 × 10^6) cells/mL. (c) The percentage of droplets containing one cell with a different initial cell concentration (N = 3 devices, n = 500 droplets). Source data are provided as a Source Data file.

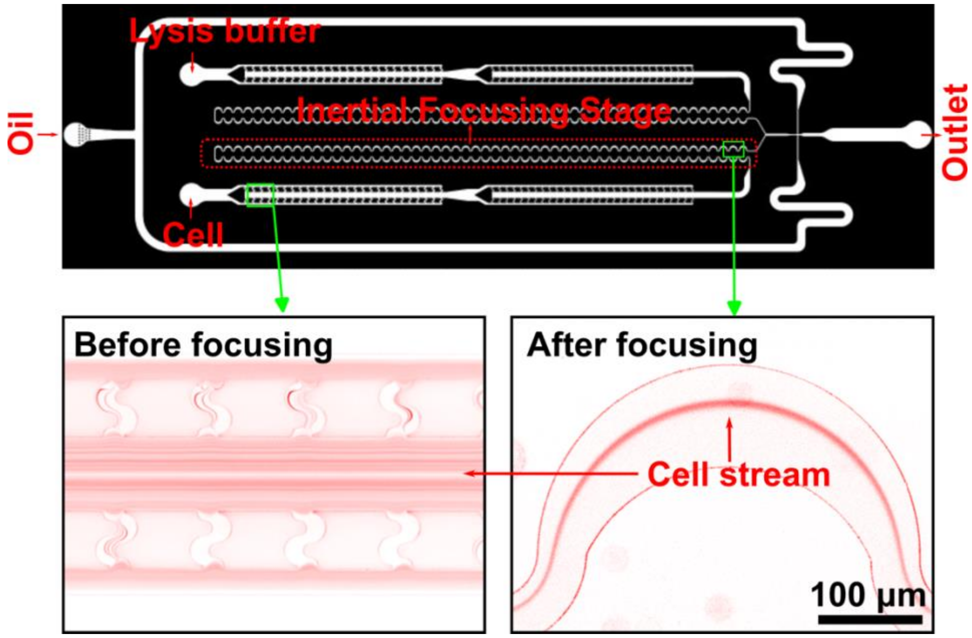

Supplementary Figure 2. **Deterministic droplet-encapsulation of inertially ordered cells**. (Top) CAD schematic of droplet-generation chip designed to utilize inertial focusing conditions to order single cells in a 'single-file queue' prior to loading cells into droplets at a target occupancy of one cell per droplet. (Bottom) Fluorescence micrographs from chip regions demarcated with green boxes (in top CAD schematic) show inertial focusing of a stream of cells expressing red fluorescence signal (left) and the downstream, ordered flow of cells prior to encapsulation in droplets (right).

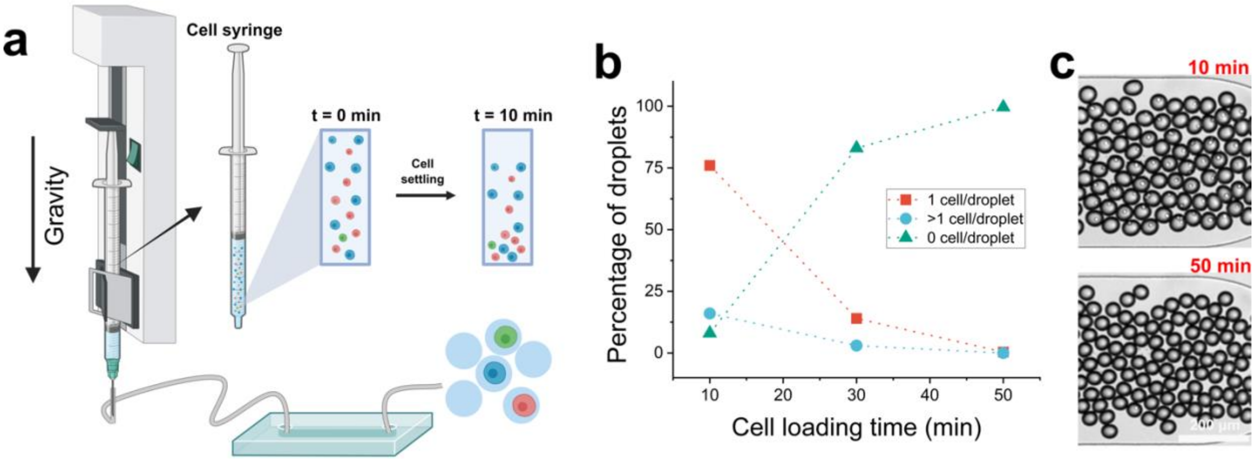

Supplementary Figure 3. Cell encapsulation under the effect of gravity. (a) Schematic illustration of droplet generation and cell encapsulation workflow. Gravity-induced cell sedimentation during the loading period results in a time-variant density of the cell suspension near the syringe outlet. Created with BioRender.com released under a Creative Commons Attribution-NonCommercial-NoDerivs 4.0 International license. (b) Cell encapsulation efficiency was measured during the loading period (N = 3 devices, n = 500 droplets). Cells: fresh MCF7; $Q_{dispersed}$ = 10 µL/min, $Q_{continuous}$ = 15 µL/min. Loading periods 10, 30, 50 min. Cell concentration: 4.0 × 10^6 cells/mL. (c) Bright-field micrographs of resultant droplet populations generated under either 10-min or 50-min cell-suspension loading periods. Source data are provided as a Source Data file.

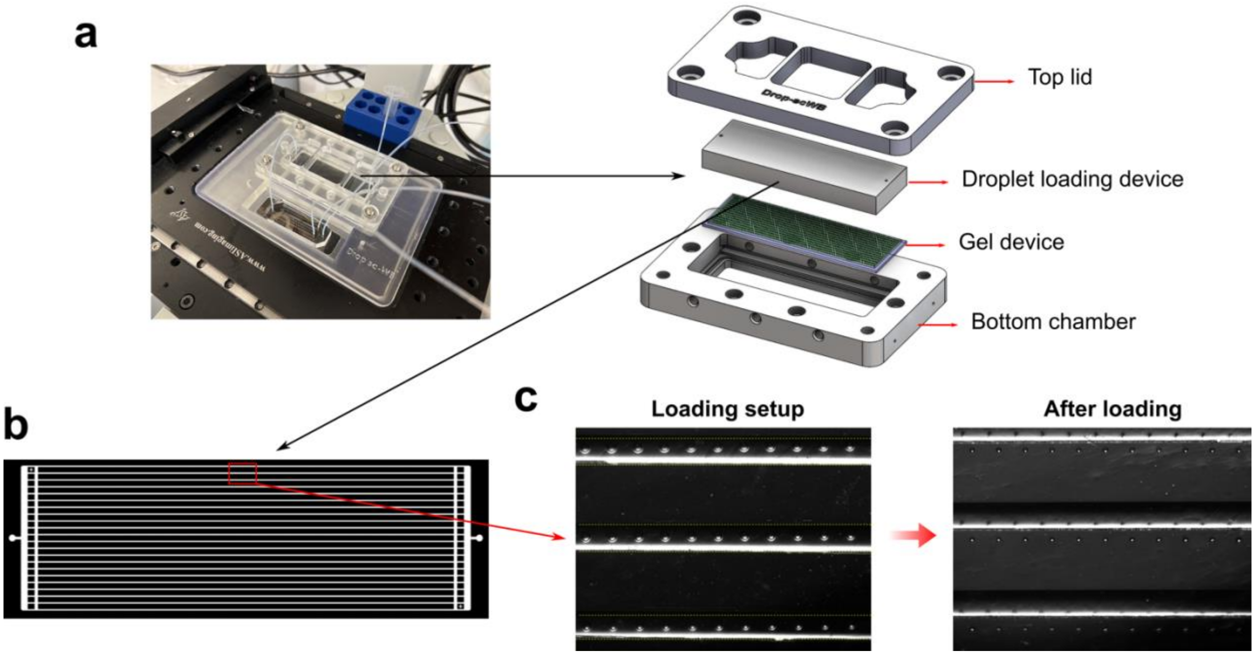

Supplementary Figure 4. All-in-one reaction holder setup and assembly. (a) overview (left) and components (right) of the all-in-one reaction holder. The top lid and bottom chamber are made of PMMA. PA-Gel device and droplet loading device are placed between the top lid and bottom chamber. (b) Top view of the droplet loading device. The microchannel consists of a series of loading channels with a width of 200 µm. (c) During droplet loading (left), the loading channels are aligned above the microwells. After the droplet loading (right), the loading device is relocated to cover the microwells.

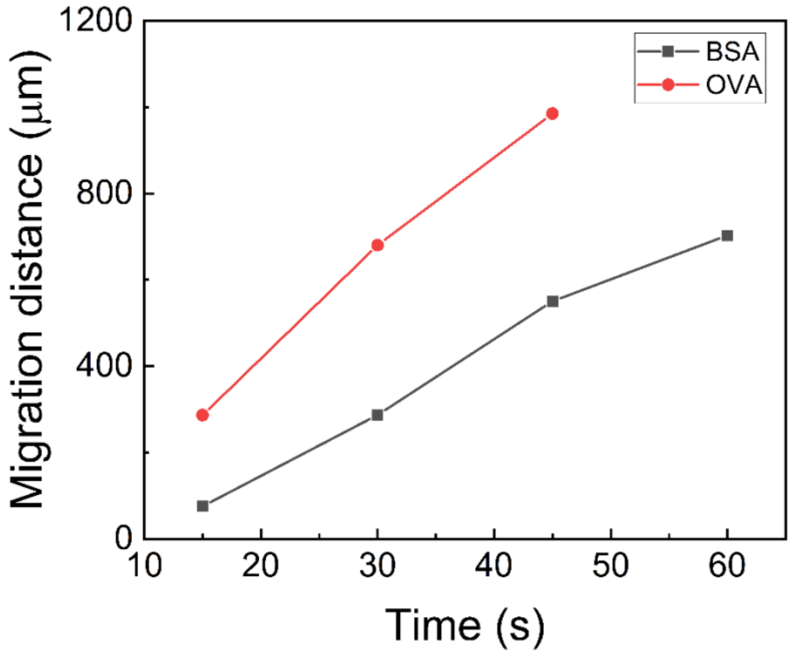

Supplementary Figure 5. Electromigration of BSA and OVA over time (experiment). *E* = 40 V/cm; Microwell diameter: 50 μm; Droplet diameter: 45 μm. Source data are provided as a Source Data file.

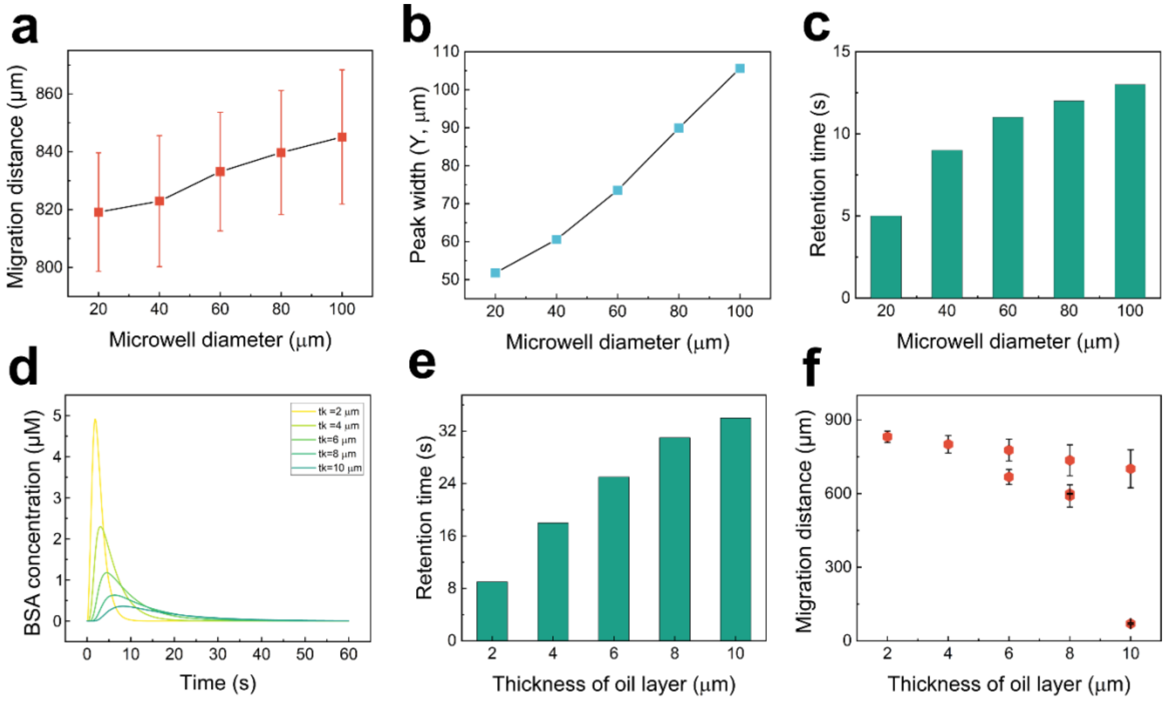

Supplementary Figure 6. Simulation of the peak center/migration distance (a), peak width (b), and retention time (c, the time all proteins migrate out of microwells) of BSA with different microwell diameters (20-100 μm). The droplet was placed at the center of the microwell, and the oil layer thickness was set to 2.5 μm. The oil layer thickness is defined as the distance between the right edge of the droplet and the left edge of the microwell. Simulation of the effect of oil thickness (tk) on the change of BSA concentration at the right edge of microwell (d), retention time (e), and peak center/migration distance of BSA (f). The error bars in (a) and (f) represent peak width (X). Source data are provided as a Source Data file.

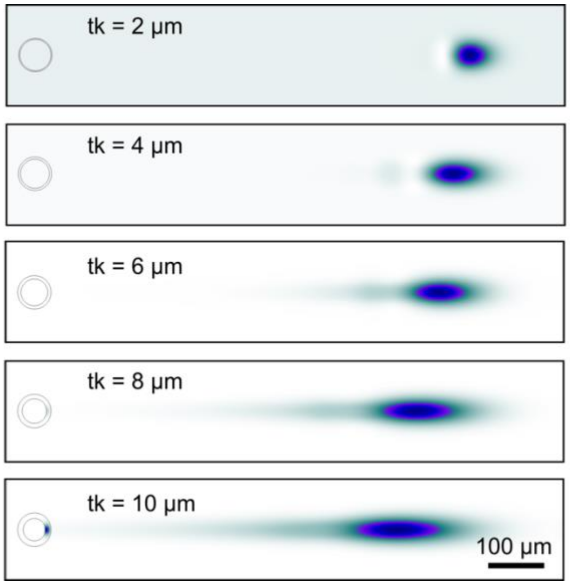

Supplementary Figure 7. Electromigration of BSA with different thicknesses (tk) of oil layer (simulation). The microwell diameter is 60 μm. E = 40 V/cm. t = 60s.

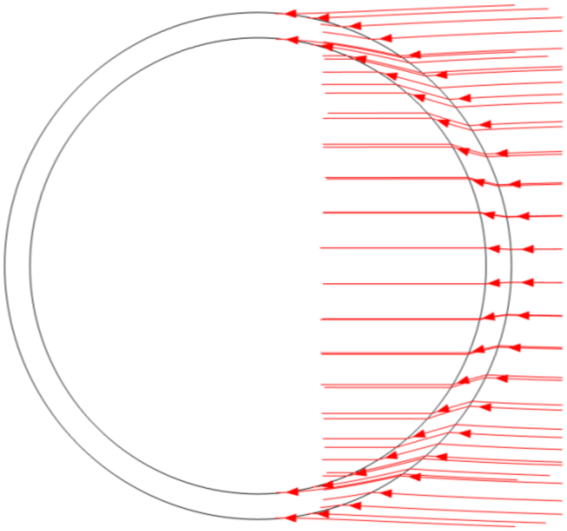

Supplementary Figure 8. Streamline plot of current density near the interface between droplet, oil, and PA-gel (simulation). The droplet (inner circle) is positioned at the center of the microwell (outer circle). E = 40 V/cm. The arrow represents the current direction.

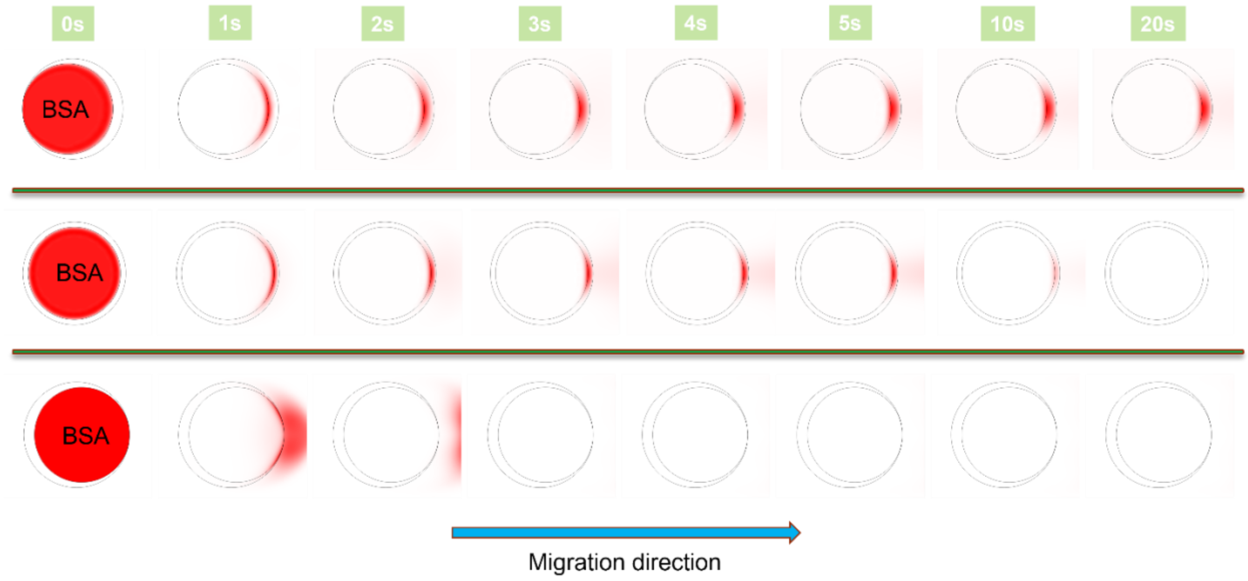

Supplementary Figure 9. Electromigration of BSA with different relative positions of droplets (simulation). From the top to the bottom panel, the relative position of the droplet is right, middle, and right to the microwell, respectively. The electric field strength is 40 V/cm, and the electrophoresis time is 0-20s.

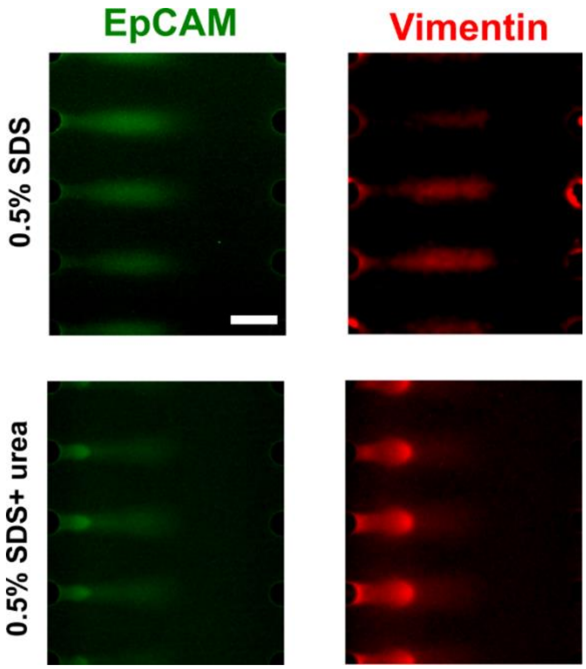

Supplementary Figure 10. Intensity profiles of EpCAM (Green, MCF7) and Vimentin (Red, MDA-MB-231) when using an antigen-retrieval buffer containing 0.5% (w/v) SDS only (top panel) and 0.5% (w/v) SDS + 6M urea (bottom panel), both after 30s electrophoresis at an electric field strength of 40V/cm. Data are representative of three independent experiments with similar results. Droplet diameter: 45 µm. Scale bar: 200 µm.

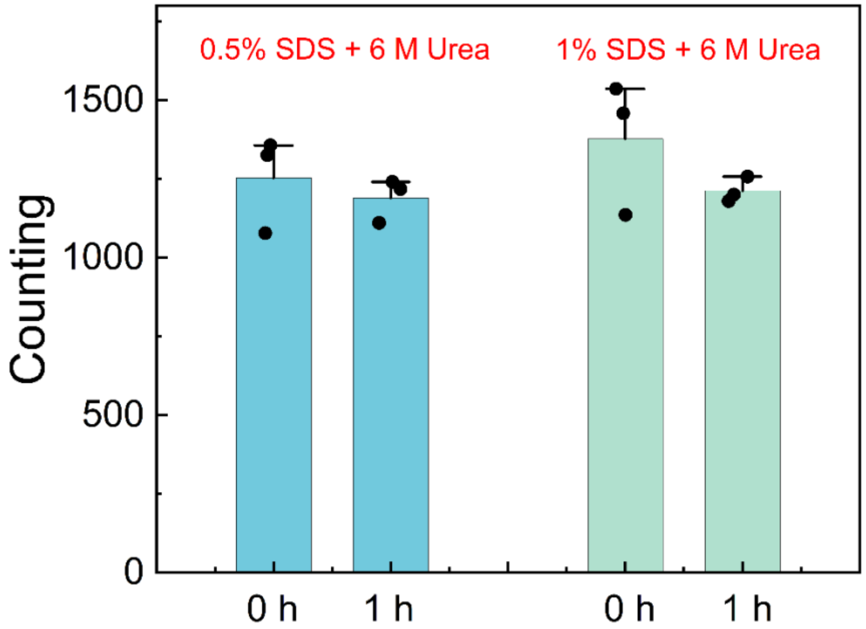

Supplementary Figure 11. Droplet enumeration after 1-hour incubation at 100ºC. Droplets are loaded with 0.5% (w/v) SDS & 6 M Urea or 1% (w/v) SDS & 6 M Urea. Droplet diameter: 45 µm. The error bars represent the standard deviation of the mean (n = 3 measurements). Source data are provided as a Source Data file.

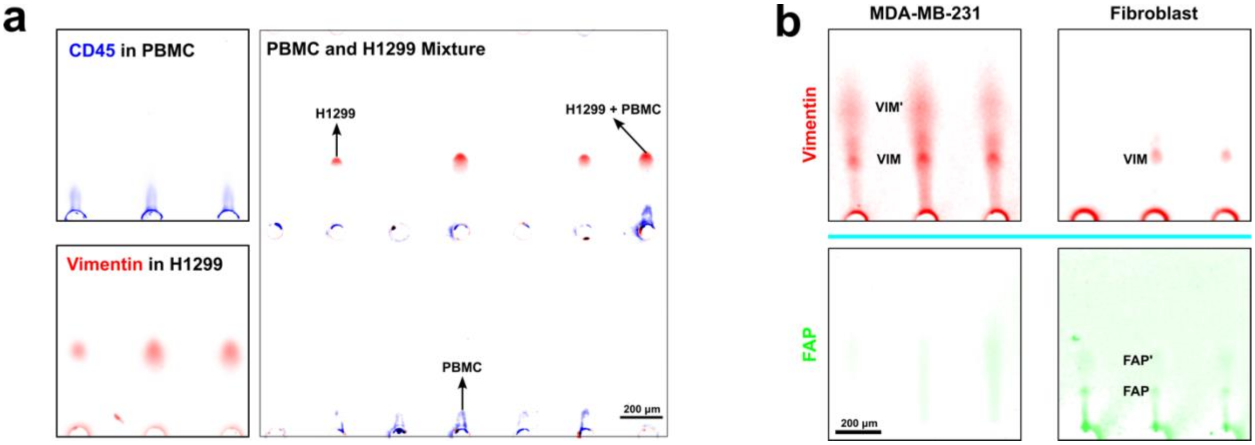

Supplementary Figure 12. Identification of cell type-based protein and proteoform markers detected by single-cell western blot (Δt_{PAGE} = 30 s; *E* = 60 V/cm). (a) Single-cell western blot micrographs report differential identification of peripheral blood mononuclear cells (PBMC) and H1299 (fresh) cells using CD45 and Vimentin markers, respectively. (b) Single-cell western blot micrographs report differential identification of MDA-MB-231 and fibroblast (fresh) cells using Vimentin and Fibroblast activation protein-alpha (FAP) markers, respectively. Data are representative of three independent experiments with similar results.

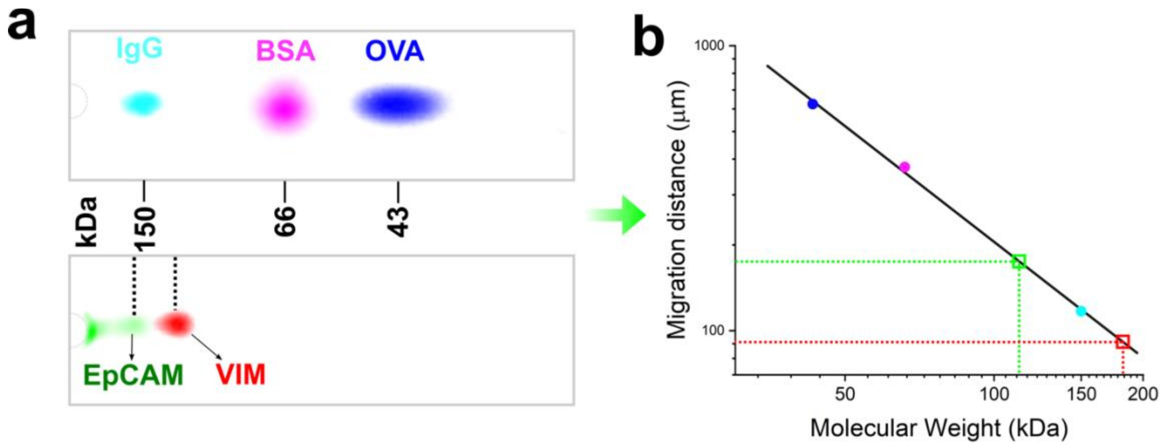

Supplementary Figure 13. Calibration of electromigration for endogenous antigen targets retrieved from PFA-fixed cells using a 3-component soluble protein ladder. (a) Fluorescence micrographs of antigen targets analyzed using the western blotting assay for DropBlot. (Top panel) Fluorescence micrograph of PAGE electromigration for a soluble 3-component protein ladder composed of AF555-BSA (pink), AF647-OVA (blue), and AF488-IgG (cyan). (Bottom panel) Fluorescence micrographs of single-cell western blotting by DropBlot for two endogenous antigen targets retrieved from PFA-fixed MDA-MB-231 cells ($\Delta t_{fixation}$ = 15 s, $\Delta t_{incubation}$ = 1.0 hr at 98ºC). (b) Molecular-mass calibration of two endogenous antigen targets retrieved from PFA-fixed cells shows the impact of PFA-chemical fixation on the physicochemical properties of protein molecules. Once retrieved from PFA-fixed cells, endogenous antigen target EpCAM spuriously (but not unexpectedly) electromigrates as a 120 kDa protein (versus known molecular mass of 30-40 kDa), and endogenous VIM spuriously (but not unexpectedly) electromigrates as a 170 kDa protein (versus known molecular mass of 57 kDa). Conditions: Δt_{PAGE} = 30 s; E = 40 V/cm. Source data are provided as a Source Data file.

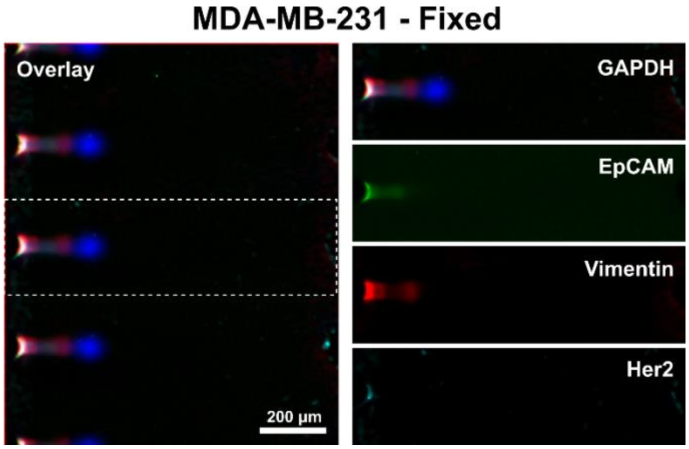

Supplementary Figure 14. Immunofluorescence image of PFA-fixed MDA-MB-231 cells. Cells were fixed with 4% PFA at room temperature for 30min. Electric field strength: 60 V/cm. Electrophoresis time: 30 s. Data are representative of three independent experiments with similar results.

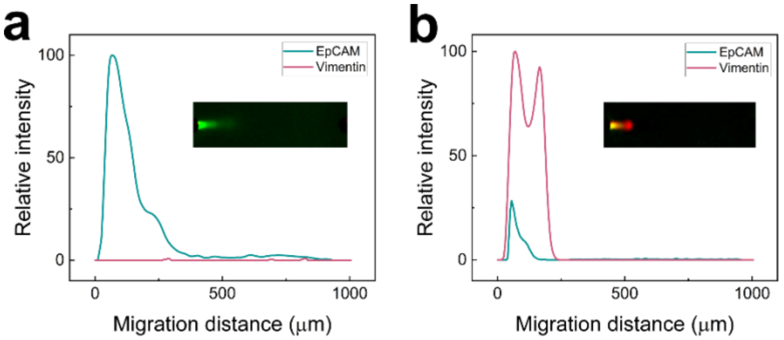

Supplementary Figure 15. Immunofluorescence image and intensity profile of EpCAM in methanol-fixed MCF7 (a) and vimentin in methanol-fixed MDA-MB-231(b). Electric field strength: 60 V/cm. Electrophoresis time: 60 s. Source data are provided as a Source Data file.

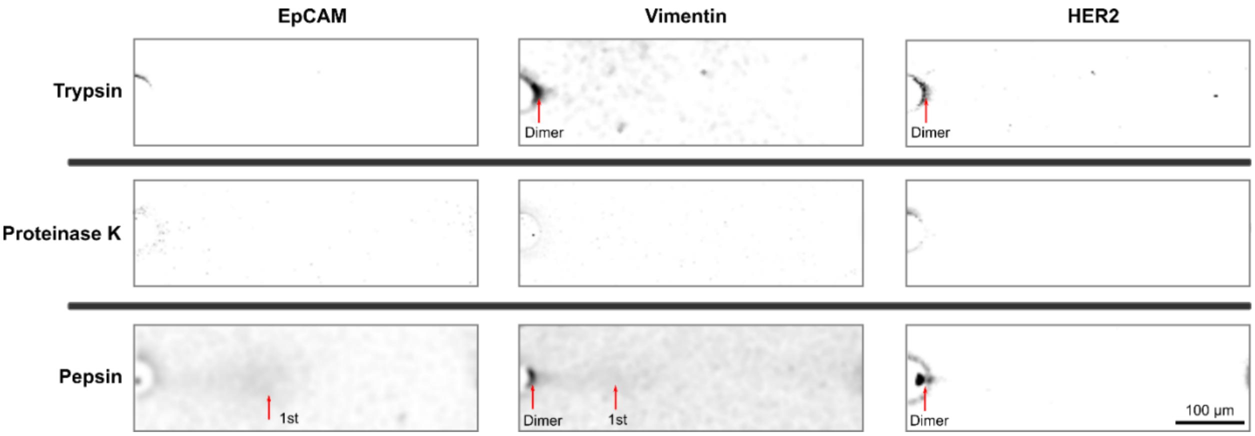

Supplementary Figure 16. Enzymatic antigen retrieval from PFA-fixed MCF7 using trypsin, proteinase K, and pepsin. The cells were fixed with 4% PFA for 15 min at room temperature. Electric field strength: 60 V/cm. Electrophoresis time: 30 s.

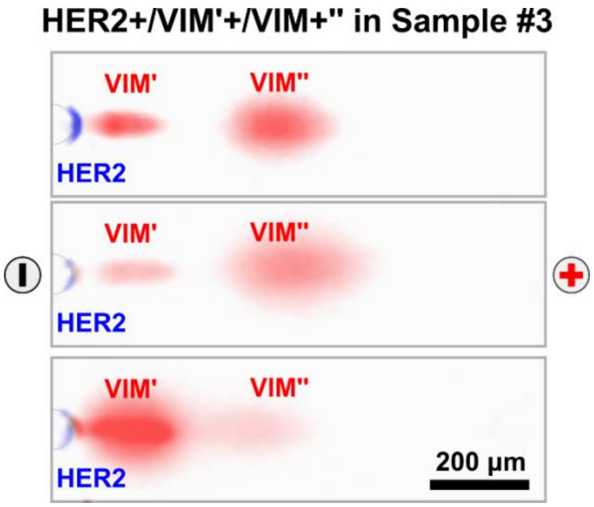

Supplementary Figure 17. Micrographs report that DropBlot detects co-expression of both VIM proteoforms (VIM’, VIM’’) in a small subpopulation of the HER2+ detection events from Patient Sample #3.

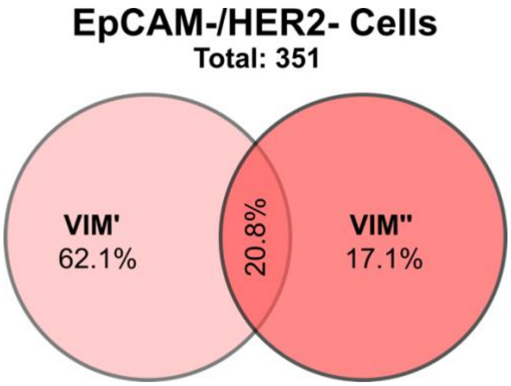

Supplementary Figure 18. Venn diagram reports the single-cell target-expression profile for PFA-fixed EpCAM-/HER2- cells (n = 351) from Sample #3 in Figure 7c.

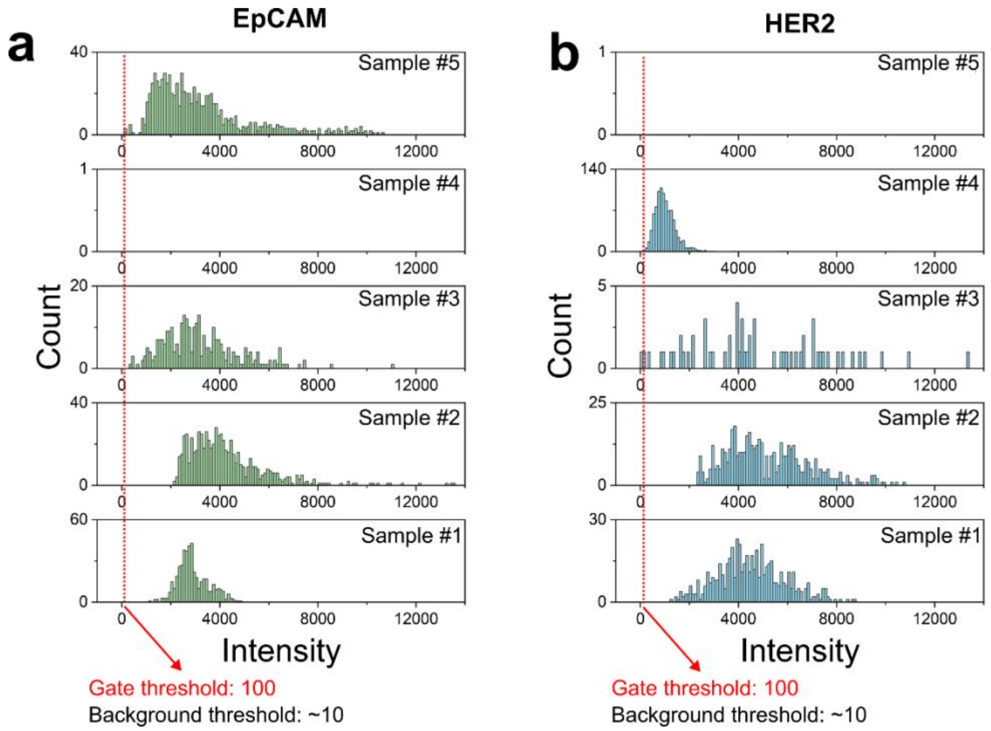

Supplementary Figure 19. Histogram of EpCAM and HER2 intensity in patient samples. Gate threshold intensity: 100; background threshold intensity: 10. Source data are provided as a Source Data file.

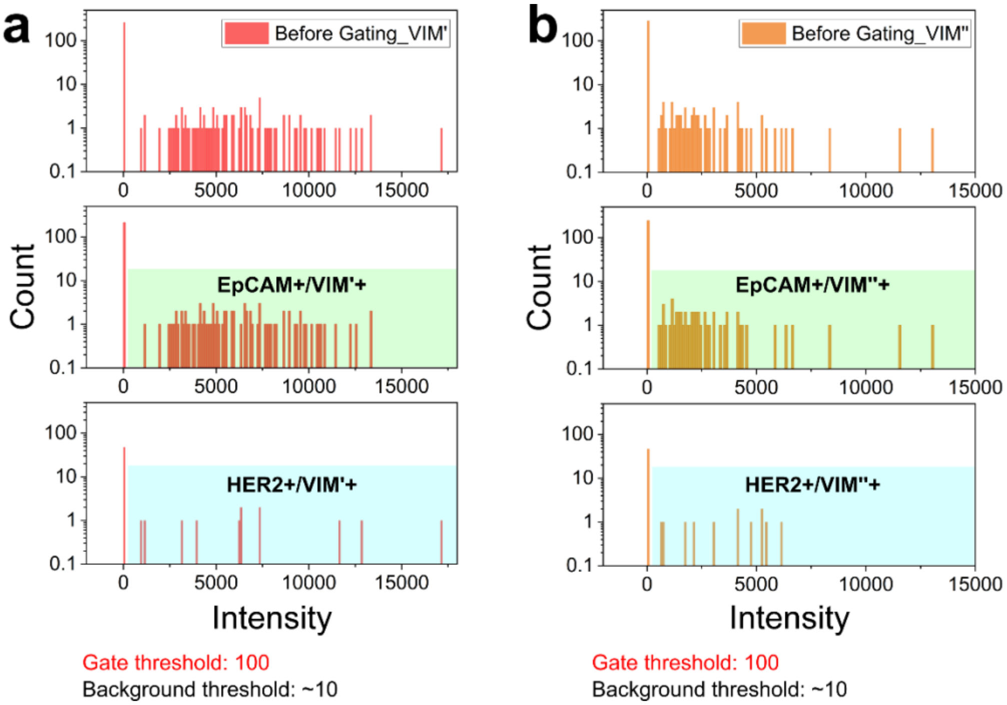

Supplementary Figure 20. Histograms of VIM’ & VIM’’ intensity before and after HER2/EpCAM gating. The light green boxes in the middle row indicate EpCAM+ cells after gating, whereas the blue boxes in the bottom row indicate HER2+ cells. Gate threshold intensity: 100; background threshold intensity: 10. Source data are provided as a Source Data file.

Supplementary Table 1: Current Single-Cell Proteomics Analysis Techniques

Name	Method	Fixed/ Live	Target	Surface /Intracellular Protein	Proteoforms detection	Throughput	Multiplexity	Pros	Cons	Ref
**Mass Spectrometry (Top down)**	Direct analysis of intact proteins	Live (fixed cells need new lysis step)	Protein	Both	Yes	<100 cells	>1000	able to intact protein molecules, high sensitivity.	Low throughput, Limited to small-to-intermediate proteins (<25 kDa); difficulty to distinguish proteoforms due to high sample complexity	1, 2, 3
**Mass Spectrometry (Bottom up)**	Direct analysis of digested proteins	Live (fixed cells need new lysis step)	Protein	Both	Yes	<100 cells	>1000	Highly multiplexed	Low throughput, limited to high abundant proteins (>10,000 copies /cell); Protein digestions will miss proteoform stoichiometry	4, 5, 6
**cyTOF (Cytometry by time of flight)**	Flow cytometry and inductive coupled plasma mass spectrometry; Use metal isotope tagged antibodies.	fixed cells, fixed tissues	Protein	both, intracellular proteins analysis requires cell fixation	Yes, based on the availability of proteoform antibody	~ 100 cells /s	~100	Highly multiplexed (>100 protein targets); Low background	Cannot be applied to live cells; Limited availability of commercial metal-isotope-labelled antibodies; difficult to analyze single cells due to low recovery rate and high sample loss.	7, 8
**FCM (flow cytometry)**	Label based, rely on fluorescent signals	live or fixed	Protein and nucleic acids	Both	Yes, based on the availability of proteoform antibody	100-10,000 cells /s	~17	high throughput, highly multiplexed	Requires large sample. Low sensitivity due to spectra overlap and autofluorescence. Limited proteoform antibodies	9, 10, 11
**IMC (Imaging Mass Cytometry**	Labeled with isotope conjugated antibodies, and analyzed with Mass Spectrometry)	Fixed / Frozen Tissue	Protein	Both	Yes	1 mm^2 / 2h	~40	High sensitivity, highly multiplexed; advance in spatial resolution	Low throughput; Limited to small proteins (<20 kDa), Limited proteoform antibodies; High Cost;	12, 13
**(PiMS) Proteoform Imaging Mass Spectrometry**	Combination of nanospray desorption electrospray (nano-DESI) and individual ion MS (I^2 MS)	Fixed Tissue	Protein	Both	Yes	2.5-4 um /s	~169 (proteoforms)	highly multiplexed; High spatial resolution	Limited to proteins smaller than 70 kDa	14
**Abseq**	label-based, antibodies are labeled with sequence tags	Live (compatible to fixed cellss)	Protein	Surface	Not applied yet, but in theory it can.	10000 cells / 1h	unlimited, but can be reduced based on the available proteins, and reading capacity)	high sensitivity to low-abundance antibodies (single molecule per cell);	some antibodies cannot be labeled with detectable tags	15
**On-Chip Cytometry (Microengraving)**	Label-Based, rely on fluorescent signals.	Live	Protein and nucleic acids	Surface	Yes	84,672 cells/ array	~4	parallel study; Cells can be recovered.	Limited to availability of proteoform antibodies.	16
**Single-cell barcode chips (SCBCs)**	Label-Based, rely on fluorescent signals.	Live/Fix	Protein	secreted proteins	Not applied yet, but in theory it can.	3000-5000 cells/ array	~42	parallel study; Cells can be recovered. Highly multiplexed	Limited to secreted proteins	17
**Quantitive Ferrohydrodynamic Cell Separation (qFCS)**	Label-based, cells are labeld with magnetic beads and sorted based on the antigen density	Live/Fix	Protein	Surface	No	30,000 cells/min	1	can detect rare cell types, as low as 10 cells/mL; high sensitivity to low abundance antigens.	Low multiplexed. Cannot analyze proteoforms.	18

**single cell western blot (scWB, 2D)**	First separate based on molecular weight, and then use fluorescent antibodies to visualize protein targets	Live	Protein	Both	Yes	~5000 cells/ array	~12	Parallel study; Capable of analyzing proteoforms.	Low multiplexed.	19, 20
**single cell western blot (scWB, 3D)**	First separate based on molecular weight, and then use fluorescent antibodies to visualize protein targets	Live	Protein	Both	Yes	2.5 cells /s, 300 cells/ array	~4	Parallel study; Low sample consumption	Low multiplexed. A large number of images to process. Cannot be applied to fixed cells	21
**Magnetic ranking cytometry (MagRC)**	Label-based, cells are labeled with magnetic nanoparticles.	Live/Fix	Protein	Surface	No	500 ul/h	1	suitable to rare cells.	Low multiplexed.	22
**Droplet-based cell screening & sorting**	Label-based, proteins are labeled with fluorescence antibodies	Live	Protein	Surface	No	2 - 5e5 cells/ h	1	high sensitivity, minimal cross-contamination	Low multiplexed.	23, 24
**Digital microfluidics (DMF)**	Digital microfluidics provide single cell sample (protein, nucleic acids) for downstream analysis (e.g., LC-MS/MS); Sample preparation is in droplet	Live/Fix	Protein/nucleic acids	Both	Not applied yet, but in theory it can.	50-500 cell / assay	>1000	High precision, low sample consumption and ability of perform complex manipulation of small volumes of liquid	Low throughput; complicated; target detection relies on other techniques (e.g., MS).	25, 26, 27
**oil-air droplet (OAD) chip**	Combination of droplet microfluidics and LC-MS/MS	Live	Protein	Both	Not applied yet, but in theory it can.	1-100 cells/ chip	~355	Low sample loss, high sample injection efficiency	Low throughput; complicated; target detection relies on other techniques (e.g, MS).	28
**Nanodroplet processing in one-pot (nanoPOTS)**	Combination of droplet microfluidics and LC-MS/MS	Live	Protein	Both	Not applied yet, but in theory it can.	10-240 cells / assay	670-3000	highly multiplexed; low sample contamination	Low throughput; complicated; target detection relies on other techniques (e.g., MS).	6, 29
**Single-cell integrated proteomic microfluidic chip (SciProChip)**	Combination of on-chip peptide preparation and LC-MS/MS	Live	Protein	Both	Not applied yet, but in theory it can.	20 / assay	~1500	highly multiplexed; low sample contamination	Low throughput; complicated; target detection relies on other techniques (e.g., MS).	30
**Immunohistochemistry (IHC) or Immunocytochemistry (ICC)**	Label-based, proteins are labeled with antibodies and visualized with colored chromogen or fluorophores	Fixed	Proteins	Both	Yes	NA	~2-50	High specificity, tissue localization, wide applicability,	Limited by the availability of antibodies, narrow dynamic range, time consuming, variability	31, 32, 33
**DropBlot**	Combination of droplet microfluidics with single-cell western blotting	Live/Fix	Protein	Both	Yes	~2,500 cells/assay	~20	Robust; capable of analyze proteoforms in fresh/fixed cells; minimized sample loss	Lysis buffer is limited due to the droplet stability. Low multiplicity; complicated	This work

Supplementary Table 2. Proteins analyzed in DropBlot

Name	MW (kDa)	Function (normal)	Locations	Proteoforms	Proteoform MW (kDa)	Proteoform Formation & Function
**GAPDH**	36	Glycolytic enzyme, regulate mRNA stability	Mainly in cytoplasm, also founded in nucleus, mitochondria, cell membrane[34, 35]	Yes, NA	NA	Not studied here
**Vimentin**	55	Structural protein, maintain cellular integrity and provide resistance against stress, cellular signal transduction[36]	Mainly in cytoplasm, can b on the surface of activated platelets (secreted by activated macrophage)[37], also found on the plasma membrane[38]	#1[39]	150, dimer	the 150 kDa band likely represents two covalently linked monomers originally belonging to adjacent dimers or tetramers that dissociate in SDS-PAGE
				#2[40]	120	a high molecular weight form of vimentin, 120 kDa, within and adjacent to vesicles near the luminal surface of Human Microvascular Endothelial Cells (HMEC)
				#3[38]	60	membrane-associated 60 kDa vimentin proteoform is also present in membranes of non-activated lymphocytes, which cannot bind extracellular anti-vimentin antibodies
				#4[41]	49	activated human T cells
				#5[42]	47	Spliced variant, ~35 amino acids smaller.
				#6[43]	32	Fragmentation pattern of vimentin due to cell apoptosis
				#7[43]	20	Fragmentation pattern of vimentin due to cell apoptosis
**EpCAM**	40	Cell adhesion protein, cell signaling, proliferation, differentiation[44]	Cell membrane	#1[45]	66, dimer	extracellular part of human EpCAM forms a heart-shaped dimer, which would form at cell surfaces
				#2[46, 47]	35	epithelium-like tumor cell lines MCF-7, T47D, and SkBR3 showed strong expression of the EpCAM protein as basic and glycosylated proteoforms of 35
				#3[48]	32	Proteolytic cleavage
				#4[48]	6	Proteolytic cleavage
**Her2**	185	Provides the cell with potent proliferative and anti-apoptosis signals[49]	Cell membrane	Yes, NA	-	Not studied here

Supplementary Table 3. Formalin vs. Methanol Fixation

Fixation Method	Formalin/PFA	Methanol
**Composition of Fixative**	Formaldehyde solution in water	Methanol
**Chemical Reaction**	Crosslinks proteins with adjacent amino acids, nucleic acids, and lipids	Lipids are removed from membranes, proteins precipitate.
**Penetration**	Slower	Faster
**Cell Morphology**	Preserve morphology well	Can cause tissue shrinkage and distortion
**Antigenicity**	May alter or mask some antigens	Generally preserve antigenicity
**Enzymatic Activity**	May preserve some enzymatic activity	Generally preserve enzymatic activity
**Nucleic Acids**	May alter or mask nucleic acids	Generally preserve nucleic acids
**Other concerns**	Protein can move through the cell during fixation, so you can see nuclear protein in cytosol	Small soluble metabolites leak.
**Applications**	Histology, immunohistochemistry, in situ hybridization, suitable to tissue	Cytology, immunofluorescence, flow cytometry, suitable to cell

Supplementary Table 4. Antigen Retrieval from 4% PFA-fixed MCF7 using Enzymes

**Enzyme**	**Trypsin**	**Trypsin**	**Trypsin**	**Trypsin**	**Trypsin**	**Trypsin**	**Proteinase K**	**Proteinase K**	**Pepsin**	**Pepsin**	**Pepsin**
**Incubation condition**	fixed cells, lysis buffe, and Trypsin were encapsulated In droplet, incubate for 30 at RT	fixed cells are incubated with trypsin solution in 1.5 mL Eppendorf tube, 30 min at RT. After incubation, wash with PBS for 3 times.	fixed cells are incubated with trypsin solution in 1.5 mL Eppendorf tube, 15 min at RT. After incubation, wash with PBS for 3 times.	fixed cells are incubated with trypsin solution in 1.5 mL Eppendorf tube, 15 min at RT. After incubation, wash with PBS for 3 times.	fixed cells are incubated with trypsin solution in 1.5 mL Eppendorf tube, 10 min at RT. After incubation, wash with PBS for 3 times.	fixed cells are incubated with trypsin solution in 1.5 mL Eppendorf tube, 5 min at RT. After incubation, wash with PBS for 3 times.	fixed cells are incubated with trypsin solution in 1.5 mL Eppendorf tube, 7 min at RT. After incubation, wash with PBS for 3 times.	fixed cells are incubated with trypsin solution in 1.5 mL Eppendorf tube, 7 min at RT. After incubation, wash with PBS for 3 times.	fixed cells are incubated with trypsin solution in 1.5 mL Eppendorf tube, 10 min at 37. After incubation, wash with PBS for 3 times.	fixed cells are incubated with trypsin solution in 1.5 mL Eppendorf tube, 10 min at 37. After incubation, wash with PBS for 3 times.	fixed cells are incubated with trypsin solution in 1.5 mL Eppendorf tube, 15 min at 37. After incubation, wash with PBS for 3 times.
**Lysis condition**	0.5%(w/v) SDS + 6M Urea, 30 min at RT	0.5%(w/v) SDS + 6M Urea, 30 min at RT	0.5%(w/v) SDS + 6M Urea, 30 min at RT	0.5%(w/v) SDS + 6M Urea, 30 min at 98C	0.5%(w/v) SDS + 6M Urea, 30 min at RT	0.5%(w/v) SDS + 6M Urea, 30 min at RT	0.5%(w/v) SDS + 6M Urea, 30 min at RT	0.5%(w/v) SDS + 6M Urea, 30 min at 98C	0.5%(w/v) SDS + 6M Urea, 30 min at RT	0.5%(w/v) SDS + 6M Urea, 30 min at 98C	0.5%(w/v) SDS + 6M Urea, 30 min at RT
**EpCAM (Y/N)**	N	N	N	N	N	N	N	N	N	N	**weak , no proteoform**
**Vimentin (Y/N)**	N	N	N	Y (Dimer)	N	N	N	N	Y	N	**Y**
**Her2 (Y/N)**	N	N	N	Y (Dimer)	N	N	N	N	Y (Dimer)	N	**Y (Dimer)**

Supplementary Table 5. Samples Tested with DropBlot

Patient	ID	ER-α Status	PR, HER2 Status	Cell Status	Type	Signal
**1**	041318	ER-α^{3+}	PR+, $HER2^{-}$	Suspension, Fresh	Invasive ductal breast tumor, PT2pN0	Yes
**2**	121715	ER-α^{1+}	PR-, $HER2^{+}$	Suspension, Fresh	Lymph node infiltrated breast tumor	Yes
**3**	041318	ER-α^{3+}	PR+, $HER2^{-}$	Tissue, Fresh	Invasive ductal breast tumor	Yes
**4**	102816	ER-α^{1+}	PR+, $HER2^{+}$	Tissue, Fresh	Triple positive breast tumor	Yes
**5**	040615	-	-	Tissue, Fresh	Cureline Bca Fresh Tissue	Yes
**6**	32818-5	ER-α^{1+}	PR+, $HER2^{-}$	Suspension, Fresh	Invasive ductal breast tumor	No
**7**	121615	ER-α^{3+}	PR^{3+}, $HER2^{-}$	Suspension, Fresh	Lymph node infiltrated breast tumor, T4bN1A	No
**8**	032918	ER-α-	PR+, $HER2^{-}$	Suspension, Fresh	Breast tumor	No
**9**	062615	-	$HER2^{+}$	FFPE	Breast tumor	No
**10**	062615	-	$HER2^{+}$	FFPE	Breast tumor	No
**11**	062615	-	$HER2^{+}$	FFPE	Breast tumor	No

Supplementary Table 6. Summary of cell subpopulations identified by DropBlot

(values represent the number of cells analyzed)

Subpopulation	VIM proteoforms	Sample #1	Sample #2	Sample #3	Sample #4	Sample #5
EpCAM+	VIM'-/VIM"-	452	501	175	0	440
	VIM'+/VIM"-	0	176	68	0	398
	VIM'-/VIM"+	0	0	37	0	0
	VIM'+/VIM"+	0	0	16	0	0
	**Total_EpCAM**	**452**	**677**	**296**	**0**	**838**
HER2+	VIM'-/VIM"-	527	383	38	817	0
	VIM'+/VIM"-	0	91	9	108	0
	VIM'-/VIM"+	0	0	10	0	0
	VIM'+/VIM"+	0	0	3	0	0
	**Total_HER2**	**527**	**474**	**60**	**925**	**0**
Other (EpCAM-/HER2-)	VIM'+/VIM"-	845	68	218	1073	604
	VIM'-/VIM"+	110	0	60	0	0
	VIM'+/VIM"+	62	0	73	0	0
	**Total_other**	**1017**	**68**	**351**	**1073**	**604**
Number of cells analyzed		**1996**	**1219**	**707**	**1998**	**1442**

References

1. van Remoortere A*, et al.* MALDI imaging and profiling MS of higher mass proteins from tissue. *J Am Soc Mass Spectrom* **21**, 1922-1929 (2010).
2. Aichler M, Walch A. MALDI Imaging mass spectrometry: current frontiers and perspectives in pathology research and practice. *Lab Invest* **95**, 422-431 (2015).
3. Do TD*, et al.* Optically Guided Single Cell Mass Spectrometry of Rat Dorsal Root Ganglia to Profile Lipids, Peptides and Proteins. *Chemphyschem* **19**, 1180-1191 (2018).
4. Budnik B, Levy E, Harmange G, Slavov N. SCoPE-MS: mass spectrometry of single mammalian cells quantifies proteome heterogeneity during cell differentiation. *Genome Biol* **19**, 161 (2018).
5. Zhu Y*, et al.* Proteomic Analysis of Single Mammalian Cells Enabled by Microfluidic Nanodroplet Sample Preparation and Ultrasensitive NanoLC-MS. *Angew Chem Int Ed Engl* **57**, 12370-12374 (2018).
6. Zhu Y*, et al.* Nanodroplet processing platform for deep and quantitative proteome profiling of 10-100 mammalian cells. *Nat Commun* **9**, 882 (2018).
7. Angelo M*, et al.* Multiplexed ion beam imaging of human breast tumors. *Nat Med* **20**, 436-442 (2014).
8. Bandura DR*, et al.* Mass cytometry: technique for real time single cell multitarget immunoassay based on inductively coupled plasma time-of-flight mass spectrometry. *Anal Chem* **81**, 6813-6822 (2009).
9. Irish JM*, et al.* B-cell signaling networks reveal a negative prognostic human lymphoma cell subset that emerges during tumor progression. *P Natl Acad Sci USA* **107**, 12747-12754 (2010).
10. Nuti E*, et al.* Bivalent Inhibitor with Selectivity for Trimeric MMP-9 Amplifies Neutrophil Chemotaxis and Enables Functional Studies on MMP-9 Proteoforms. *Cells-Basel* **9**, (2020).
11. Krutzik PO, Crane JM, Clutter MR, Nolan GP. High-content single-cell drug screening with phosphospecific flow cytometry. *Nat Chem Biol* **4**, 132-142 (2008).
12. Jackson HW*, et al.* The single-cell pathology landscape of breast cancer. *Nature* **578**, 615-+ (2020).
13. Baharlou H, Canete NP, Cunningham AL, Harman AN, Patrick E. Mass Cytometry Imaging for the Study of Human Diseases-Applications and Data Analysis Strategies. *Front Immunol* **10**, (2019).
14. Su P*, et al.* Highly multiplexed, label-free proteoform imaging of tissues by individual ion mass spectrometry. *Sci Adv* **8**, eabp9929 (2022).
15. Shahi P, Kim SC, Haliburton JR, Gartner ZJ, Abate AR. Abseq: Ultrahigh-throughput single cell protein profiling with droplet microfluidic barcoding. *Sci Rep* **7**, 44447 (2017).
16. Ogunniyi AO*, et al.* Profiling human antibody responses by integrated single-cell analysis. *Vaccine* **32**, 2866-2873 (2014).
17. Lu Y*, et al.* Highly multiplexed profiling of single-cell effector functions reveals deep functional heterogeneity in response to pathogenic ligands. *Proc Natl Acad Sci U S A* **112**, E607-615 (2015).
18. Liu Y, Vieira RMS, Mao L. Simultaneous and Multimodal Antigen-Binding Profiling and Isolation of Rare Cells via Quantitative Ferrohydrodynamic Cell Separation. *ACS Nano* **17**, 94-110 (2023).
19. Hughes AJ, Spelke DP, Xu Z, Kang CC, Schaffer DV, Herr AE. Single-cell western blotting. *Nat Methods* **11**, 749-755 (2014).
20. Sinkala E*, et al.* Profiling protein expression in circulating tumour cells using microfluidic western blotting. *Nat Commun* **8**, 14622 (2017).
21. Grist SM, Mourdoukoutas AP, Herr AE. 3D projection electrophoresis for single-cell immunoblotting. *Nat Commun* **11**, 6237 (2020).
22. Poudineh M*, et al.* Tracking the dynamics of circulating tumour cell phenotypes using nanoparticle-mediated magnetic ranking. *Nat Nanotechnol* **12**, 274-281 (2017).

23. Mazutis L, Gilbert J, Ung WL, Weitz DA, Griffiths AD, Heyman JA. Single-cell analysis and sorting using droplet-based microfluidics. *Nat Protoc* **8**, 870-891 (2013).
24. Martino C, Zagnoni M, Sandison ME, Chanasakulniyom M, Pitt AR, Cooper JM. Intracellular protein determination using droplet-based immunoassays. *Anal Chem* **83**, 5361-5368 (2011).
25. Eyer K*, et al.* Single-cell deep phenotyping of IgG-secreting cells for high-resolution immune monitoring. *Nat Biotechnol* **35**, 977-982 (2017).
26. Steinbach MK, Leipert J, Blurton C, Leippe M, Tholey A. Digital Microfluidics Supported Microproteomics for Quantitative Proteome Analysis of Single Caenorhabditis elegans Nematodes. *J Proteome Res* **21**, 1986-1996 (2022).
27. Leipert J, Steinbach MK, Tholey A. Isobaric Peptide Labeling on Digital Microfluidics for Quantitative Low Cell Number Proteomics. *Anal Chem* **93**, 6278-6286 (2021).
28. Li ZY*, et al.* Nanoliter-Scale Oil-Air-Droplet Chip-Based Single Cell Proteomic Analysis. *Anal Chem* **90**, 5430-5438 (2018).
29. Cong Y*, et al.* Ultrasensitive single-cell proteomics workflow identifies >1000 protein groups per mammalian cell. *Chem Sci* **12**, 1001-1006 (2020).
30. Gebreyesus ST*, et al.* Streamlined single-cell proteomics by an integrated microfluidic chip and data-independent acquisition mass spectrometry. *Nat Commun* **13**, 37 (2022).
31. Ramos-Vara JA. Technical aspects of immunohistochemistry. *Vet Pathol* **42**, 405-426 (2005).
32. Happonen RP, Heikinheimo K. Introduction to immunocytochemistry. *Proc Finn Dent Soc* **85**, 61-67 (1989).
33. Hofman P*, et al.* Multiplexed Immunohistochemistry for Molecular and Immune Profiling in Lung Cancer-Just About Ready for Prime-Time? *Cancers (Basel)* **11**, (2019).
34. Nicholls C, Li H, Liu JP. GAPDH: a common enzyme with uncommon functions. *Clin Exp Pharmacol Physiol* **39**, 674-679 (2012).
35. Tristan C, Shahani N, Sedlak TW, Sawa A. The diverse functions of GAPDH: views from different subcellular compartments. *Cell Signal* **23**, 317-323 (2011).
36. Ivaska J, Pallari HM, Nevo J, Eriksson JE. Novel functions of vimentin in cell adhesion, migration, and signaling. *Exp Cell Res* **313**, 2050-2062 (2007).
37. Mor-Vaknin N, Punturieri A, Sitwala K, Markovitz DM. Vimentin is secreted by activated macrophages. *Nat Cell Biol* **5**, 59-63 (2003).
38. Bilalic S*, et al.* Lymphocyte activation induces cell surface expression of an immunogenic vimentin isoform. *Transpl Immunol* **27**, 101-106 (2012).
39. Monico A, Guzman-Caldentey J, Pajares MA, Martin-Santamaria S, Perez-Sala D. Molecular Insight into the Regulation of Vimentin by Cysteine Modifications and Zinc Binding. *Antioxidants-Basel* **10**, (2021).
40. Xu B, deWaal RM, Mor-Vaknin N, Hibbard C, Markovitz DM, Kahn ML. The endothelial cell-specific antibody PAL-E identifies a secreted form of vimentin in the blood vasculature. *Mol Cell Biol* **24**, 9198-9206 (2004).
41. Rose ML. Role of anti-vimentin antibodies in allograft rejection. *Hum Immunol* **74**, 1459-1462 (2013).
42. von Brandenstein M*, et al.* Vimentin 3, the New Hope, Differentiating RCC versus Oncocytoma. *Dis Markers* **2015**, (2015).
43. Mary S, Kulkarni MJ, Mehendale SS, Joshi SR, Giri AP. Differential accumulation of vimentin fragments in preeclamptic placenta. *Cytoskeleton* **74**, 420-425 (2017).
44. Huang L*, et al.* Functions of EpCAM in physiological processes and diseases (Review). *Int J Mol Med* **42**, 1771-1785 (2018).
45. Pavsic M, Guncar G, Djinovic-Carugo K, Lenarcic B. Crystal structure and its bearing towards an understanding of key biological functions of EpCAM. *Nature Communications* **5**, (2014).

46. Martowicz A, Spizzo G, Gastl G, Untergasser G. Phenotype-dependent effects of EpCAM expression on growth and invasion of human breast cancer cell lines. *Bmc Cancer* **12**, (2012).
47. Schmidt DS, Klingbeil P, Schnolzer M, Zoller M. CD44 variant isoforms associate with tetraspanins and EpCAM. *Experimental Cell Research* **297**, 329-347 (2004).
48. Blazar M*, et al.* Epidermal growth factor-like repeats mediate lateral and reciprocal interactions of Ep-CAM molecules in homophilic adhesions. *Mol Cell Biol* **21**, 2570-2580 (2001).
49. Gutierrez C, Schiff R. HER2: biology, detection, and clinical implications. *Archives of pathology & laboratory medicine* **135**, 55-62 (2011).
