# Supplementary material for: DropBlot: single-cell western blotting of chemically fixed cancer cells
Source: Nat Commun. 2024 Jul 13;15:5888. doi: 10.1038/s41467-024-50046-0 (PMC11246512; doi:10.1038/s41467-024-50046-0)
Supplement: Supplementary file 3 — Reporting summary [file 41467_2024_50046_MOESM3_ESM.pdf]

nature portfolio

Corresponding author(s): *Amy E. Herr*

Last updated by author(s): 06/22/2024

Reporting Summary

Nature Portfolio wishes to improve the reproducibility of the work that we publish. This form provides structure for consistency and transparency in reporting. For further information on Nature Portfolio policies, see our Editorial Policies and the Editorial Policy Checklist.

Statistics

For all statistical analyses, confirm that the following items are present in the figure legend, table legend, main text, or Methods section.

n/a	Confirmed	
☐	☒	The exact sample size (*n*) for each experimental group/condition, given as a discrete number and unit of measurement
☐	☒	A statement on whether measurements were taken from distinct samples or whether the same sample was measured repeatedly
☒	☐	The statistical test(s) used AND whether they are one- or two-sided<br>*Only common tests should be described solely by name; describe more complex techniques in the Methods section.*
☒	☐	A description of all covariates tested
☒	☐	A description of any assumptions or corrections, such as tests of normality and adjustment for multiple comparisons
☐	☒	A full description of the statistical parameters including central tendency (e.g. means) or other basic estimates (e.g. regression coefficient) AND variation (e.g. standard deviation) or associated estimates of uncertainty (e.g. confidence intervals)
☒	☐	For null hypothesis testing, the test statistic (e.g. *F*, *t*, *r*) with confidence intervals, effect sizes, degrees of freedom and *P* value noted<br>*Give P values as exact values whenever suitable.*
☒	☐	For Bayesian analysis, information on the choice of priors and Markov chain Monte Carlo settings
☒	☐	For hierarchical and complex designs, identification of the appropriate level for tests and full reporting of outcomes
☒	☐	Estimates of effect sizes (e.g. Cohen's *d*, Pearson's *r*), indicating how they were calculated

Our web collection on statistics for biologists contains articles on many of the points above.

Software and code

Policy information about availability of computer code

Data collection	*COMSOL 5.4 to simulate the protein electromigration; MetaMorph and GenePix to take fluorescent images*
Data analysis	*MATLAB 2021 to analyze images*

For manuscripts utilizing custom algorithms or software that are central to the research but not yet described in published literature, software must be made available to editors and reviewers. We strongly encourage code deposition in a community repository (e.g. GitHub). See the Nature Portfolio guidelines for submitting code & software for further information.

Data

Policy information about availability of data

All manuscripts must include a data availability statement. This statement should provide the following information, where applicable:

- Accession codes, unique identifiers, or web links for publicly available datasets
- A description of any restrictions on data availability
- For clinical datasets or third party data, please ensure that the statement adheres to our policy

The data supporting the conclusions of this work in the main text or the Supplementary Information are available from the corresponding authors upon request. Custom MATLAB script and key COMSOL parameters are deposited in a GitHub repository: https://doi.org/10.5281/zenodo.11437115; Source data for each figure has been uploaded to figshare (https://figshare.com/s/cc9d8b06fa59fa23348c)

Research involving human participants, their data, or biological material

Policy information about studies with human participants or human data. See also policy information about sex, gender (identity/presentation), and sexual orientation and race, ethnicity and racism.

Reporting on sex and gender	Samples were procured from the Stanford Cancer Institute's Tissue Procurement Shared Resource repository. The authors have no access to sex and gender information. This study focuses on protein analysis within the collected samples rather than examining differences between sexes.
Reporting on race, ethnicity, or other socially relevant groupings	No analysis were performed according by race or ethnicity. There were no specific inclusion criteria based on race, ethnicity during the sample selection process.
Population characteristics	Samples from patient diagnosed with breast cancer
Recruitment	Patients were recruited through their oncologists. Not all approached patients chose to participate in the sample collection, introducing potential self-selection bias. However, the authors believe it is unlikely that this would result in a bias in any biological variable.
Ethics oversight	This research complies with all relevant ethical guidelines. Human tissues used in this study were obtained as de-identified samples from the Stanford Cancer Institute's Tissue Procurement Shared Resource repository and are not considered human subjects as defined by 45 CFR 46, and thus are not regulated by the Office of Human Subjects Protections nor subject to institutional review board (IRB) oversight, as the authors or sponsors have no access to identifying information.

Note that full information on the approval of the study protocol must also be provided in the manuscript.

Field-specific reporting

Please select the one below that is the best fit for your research. If you are not sure, read the appropriate sections before making your selection.

☒ Life sciences ☐ Behavioural & social sciences ☐ Ecological, evolutionary & environmental sciences

For a reference copy of the document with all sections, see nature.com/documents/nr-reporting-summary-flat.pdf

Life sciences study design

All studies must disclose on these points even when the disclosure is negative.

Sample size	The sample (cell) size utilized in this study spanned from 100 to 2500 cells per experiment. The selection of sample size in this instance was constrained by the capacity of the microfluidic device (2500 cells maximum) specifically designed for this research.
Data exclusions	We use polyacrylamide gel device patterned with microwells. The cell signal from broken or edge area will be discard. Fluorescent Signal without Gaussian distribution will be removed.
Replication	Experiment will be repeated for 3 times using different devices. ~2500 microwells on the same device are independent and will not affect each other
Randomization	*Prior to the experiment, cells were thoroughly resuspended, and an appropriate sample amount was randomly allocated for the experiment.*
Blinding	We are not blind to the data collection/analysis, as all the data will be evaluated.

Reporting for specific materials, systems and methods

We require information from authors about some types of materials, experimental systems and methods used in many studies. Here, indicate whether each material, system or method listed is relevant to your study. If you are not sure if a list item applies to your research, read the appropriate section before selecting a response.

Materials & experimental systems

n/a	Involved in the study
☐	☒ Antibodies
☐	☒ Eukaryotic cell lines
☒	☐ Palaeontology and archaeology
☒	☐ Animals and other organisms
☒	☐ Clinical data
☒	☐ Dual use research of concern
☒	☐ Plants

Methods

n/a	Involved in the study
☒	☐ ChIP-seq
☒	☐ Flow cytometry
☒	☐ MRI-based neuroimaging

Antibodies

Antibodies used

mouse anti-ErbB2/Her2 (Abcam, cat. no. AB16901, RRID: AB_443537), rabbit anti-EpCAM (Abcam, cat. no. AB32392, RRID: AB_732181), mouse anti-Vimentin (Abcam, cat.no. AB8978, RRID: AB_306907), rabbit anti-Vimentin (Abcam, ab92547) and goat anti-GAPDH (Sigma-Aldrich, cat. no. SAB2500450, AB_10603419); Anti-CD45 (Abcam, ab8216), Anti-FAP (Abcam, ab207178) ; rTurboGFP (GFP, Evrogen, Russia, FP552). • Secondary antibodies: Alexa Fluor 488 donkey anti-rabbit IgG (Thermo Fisher Scientific, cat. no. A21206, RRID: AB_2535792), Alexa Fluor 555 donkey anti-goat (Thermo Fisher Scientific, cat. no. A21432, RRID: AB_2535853), Alexa Fluor 594 donkey anti-mouse (Thermo Fisher Scientific, cat. no. A21203, RRID: AB_141633), Alexa Fluor 647 donkey anti-mouse (Thermo Fisher Scientific, cat. no. A32787, RRID: AB_2762830)

Validation

All antibodies are pre-validated by the vendors (Abcam or Sigma Aldrich or thermofisher). In this research, we used fluorescent signal of secondary antibodies to verify the binding of antibodies.

Eukaryotic cell lines

Policy information about cell lines and Sex and Gender in Research

Cell line source(s)

Human breast cancer cell lines, including MCF7, MCF7/GFP, and MDA-MB-231/GFP were obtained from obtained from the UC Berkeley Tissue Culture Facility. Human lung cancer cell line (H1299, CRL-5803), and Fibroblasts (PCS-201-012) were purchased from ATCC. PBMCs were prepared from whole blood purchased from Zen-Bio (SER-WB-SDS)

Authentication

All cell lines were tested by ATCC

Mycoplasma contamination

Cell lines were tested by UC Berkeley QB3 Cell & Tissue Analysis Facility or ATCC.

Commonly misidentified lines
(See ICLAC register)

NA

Plants

Seed stocks

NA

Novel plant genotypes

NA

Authentication

NA
